# Renal artery Doppler in fetal sonography: A narrative review

**DOI:** 10.18502/ijrm.v21i10.14534

**Published:** 2023-11-24

**Authors:** Ashraf Sadat Jamal, Mahdieh Modarresi

**Affiliations:** Department of Obstetrics and Gynecology, Shariati Hospital, Tehran University of Medical Sciences, Tehran, Iran.

**Keywords:** Ultrasonography, Doppler, Renal artery, Fetal monitoring.

## Abstract

Doppler imaging is a non-invasive method in evaluating fetal circulation. Renal artery doppler (RAD) has been used for assessing fetal well-being in several studies. The aim of this narrative review was to accumulate and classify current evidence on RAD in fetal sonography. Articles until November 2022 were searched. After removing ineligible articles, 51 studies were included. Present articles were about RAD assessment in cases with amniotic fluid level changes, fetal growth restriction, fetal renal diseases, monochorionic twin pregnancies, preeclampsia, and gestational diabetes mellitus. The complex physiology of fetal kidney function may explain different results observed in different studies about the role of RAD in fetal assessment. It seems this factor can be useful in assessing some groups like diabetic pregnant women, and it should be used accompanying other related factors like kidney size. Further research is needed to evaluate the effectiveness of RAD in clinical management.

## 1. Introduction

Detection of fetal risk is an important issue in today's obstetrics. Ultrasound plays a significant role, and doppler imaging is a non-invasive method in the assessment of fetal circulation. Several studies have evaluated fetal doppler imaging, most of them studying uterine artery, umbilical artery, and middle cerebral artery, but doppler study of other vessels like renal artery (RA) can also be useful in assessing fetal well-being. Current guidelines do not recommend RA doppler (RAD) as a surveillance tool in fetal sonography, but its clinical effectiveness has been reported in several articles, and an association with adverse perinatal outcomes has been suggested. RAD was applied for monitoring high-risk pregnancies, especially those with altered distribution of blood within the fetus. It is known that when a fetus is compromised, more blood goes toward the vital organs than to the nonessential organs like kidneys (1, 2).

In addition, it can show the function of fetal kidneys and aids in the diagnosis of fetal renal pathologies. Renal urine production is dependent on renal blood flow, and vascular alterations are known to play an important role in reducing urine output. Measurement of RA resistance may therefore be used to indirectly assess decline in renal function in early stages. This technique may possess a higher degree of precision in comparison to the measurement of amniotic fluid level. The latter is influenced by various mechanisms apart from renal urine production, such as mechanical obstruction of the urinary tract (3). Moreover, due to the low sensitivity of Doppler studies as a prenatal diagnostic test, combining multiple tests, including RAD, can help better evaluation of the fetus's condition.

Articles until November 2022 were searched. After removing ineligible articles, 51 studies about RAD were included. The review aimed to accumulate and classify current evidence on RAD in fetal sonography and provide recommendations for future research.

## 2. Assessment of fetal RAD

**Figure 1:**
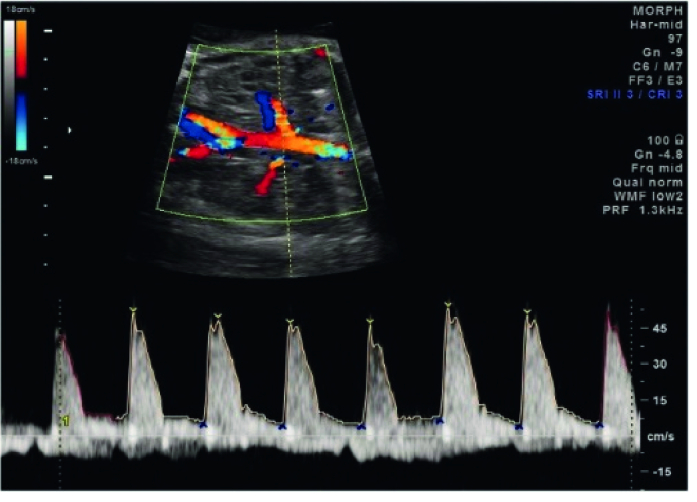
Fetal renal artery doppler.

The method of assessment of RAD is nearly the same in the studies. According to literature, after obtaining a coronal view of the fetal kidneys, color flow Doppler is used to see the RA from the aorta to the kidney (4). A 2-3 mm sample gate and a low-wall filter of around 30-60 Hertz is set. Both fetal RAs were assessed (Figure 1).

One of the studies investigated the pulsatility/resistance indices and absolute velocities of blood flow in fetal RAs on both sides and in 2 sites of each vessel. Mean values of the pulsatility index (PI) and resistive index (RI) in the right and left RAs and at the proximal and distal were found to be similar. Mean peak systolic value (PSV) and end-diastolic value were more at the proximal site on both sides. They declared that it is important to determine the exact site of velocity measurements in fetal RAs (5).

In a survey evaluating the inter-observer reliability of Doppler assessment in pregnancies of 25-39 wk gestation, 2 persons measured PSV, minimum diastolic, mean velocities, PI, and RI from the middle cerebral and renal arteries. Inter-observer agreement was seen for PI and minimum diastolic velocity in both the arteries. They suggested these 2 measurements as more appropriate to use (6).

## 3. Normal ranges of fetal RA blood flow

Quantifying the normal changes of the fetal renal blood flow in pregnancy enables identifying blood flow abnormalities, such as what happens in fetal hypoxia and how blood is diverted from the kidneys to more essential organs. Assessing RI and PI of the fetal RA may be a more sensitive way to evaluate renal hemodynamics and can help in diagnosing renal diseases.

In a survey, serial ultrasound examinations were done in 16-38 wk gestation uncomplicated pregnancies to develop standard charts of normal ranges for RA indices for application in clinical practice (4). No significant difference was observed in right and left kidneys or in gender for RI or PI. Little change was observed in these indices during pregnancy. They suggested that the little change in renal hemodynamics during pregnancy is probably because of the limited function of fetal kidneys, as the placenta does the main prenatal excretory function, and a low fraction of cardiac output goes to fetal kidneys. They thought changes in RA blood flow, might become a good method to diagnose fetal growth restriction (FGR) and renal blood flow indices may be able to show the severity of the problem. This marker can predict renal function after delivery, especially in renal diseases. In the same study, values for PI demonstrated minimal alterations throughout the pregnancy, while the PSV values showed an increasing trend as gestational age increased until term (7).

In a prospective longitudinal survey on women who had appropriately grown gestational age fetuses between 24 and 38 wk, nomograms for 5 vessels including RA were made. In RA, the indices had minimal variation during gestation and this artery stayed highly resistant during pregnancy (2). On the other hand, a study on a large population of 1556 healthy fetuses, providing reference nomograms for PI as well as for several ratios between its values in peripheral and cerebral vessels, showed the RA-PI significantly decreases during gestation with a linear regression pattern (8).

## 4. Fetal RAD an alternative to amniotic fluid level changes

Sonography is used to measure the amount of amniotic fluid in order to assess fetal urine production and kidney function. However, the ultrasound measurement of amniotic fluid is not completely reliable because the methods for measuring the amniotic fluid do not closely relate to the actual volume. It is important to note that amniotic fluid levels can be altered by factors other than changes in fetal urine production. For example, obstruction of the fetal gastrointestinal tract can lead to polyhydramnios, while obstruction of the lower urinary system can lead to oligo/anhydramnios. Renal filtration and urine production depend on the arterial perfusion of the kidney, therefore RAD parameters may serve as a more appropriate indicator of fetal kidney function. Several studies have investigated the correlation between fetal RA indices and amniotic fluid levels; however, they have reached conflicting results (3) (Table I).

A study assessing the hourly urine output of the fetus by sonographic measurement of fetal bladder showed that this parameter had a significant correlation with the RI of RA and umbilical artery (9). Another study investigating the hemodynamic changes of the fetal RA in isolated oligohydramnios showed that the RI was the most accurate Doppler parameter relevant to isolated oligohydramnios (10). A survey comparing the changes in amniotic fluid index (AFI) with the changes in the PI of the fetal vessels detected a notable inverse relationship between the alteration in AFI and the alteration in RA-PI during pregnancy. As the AFI increased, the RA-PI decreased, even after controlling for gestational age (11). A study evaluating renal and umbilical artery Doppler values in low-risk pregnancies, showed that RA-PI values were elevated in pregnant women with oligohydramnios and increased in early pregnancy prior to oligohydramnios development (12).

2 studies on post-term pregnancies, showed increased renal arterial impedance in the presence of oligohydramnios, probably because of the association between redistribution of fetal arterial blood flow with oligohydramnios. RAD was a better predictor of oligohydramnios compared to the umbilical artery (13, 14). Another article on prolonged pregnancies found a significant relationship between the AFI and the fetal renal systolic/diastolic ratio as well (15).

In contrast to the studies mentioned, such an association was not seen in some other articles. In one of them significantly lower fetal RA-PI was seen in the borderline AFI group in comparison to the normal AFI group and their results indicated that borderline AFI was not a risk for perinatal complications. They supposed reducing amniotic fluid in these patients may be due to insufficient maternal fluid intake and related decreasing blood flow due to maternal vascular volume (16). Isolated oligohydramnios, considered as a marker of chronic fetal hypoxemia or poor placental functions were assessed in an article that found no difference in MoM of RA-PI values between oligohydramnios and normal groups (17). Also, another study showed that RA-PI was not significantly different between pregnancies with isolated oligohydramnios and normal amniotic fluid, assuming that isolated oligohydramnios may not be related to impairment of RA blood flow (18). Similarly, another research also saw no significant correlations between AFI and PI or PSV among low-risk pregnancies and assumed renal blood flow disorders are not expected in a low-risk population (7).

A group of researchers thought the conflict in different studies about the association of RA-PI, and AFI is because the size of the vascular bed and the organ affects the resistance of vasculature. They corrected RA resistance indices for the size of the kidney and a new index (volume-corrected RA PI) was achieved by dividing the RA-PI by the renal volume. Their results showed that this index predicts oligohydramnios better than the uncorrected and gestational age-adjusted RA-PI. Their new index could not predict polyhydramnios (3). In another study, no statistically significant difference was observed in the fetal RA-PI of the patients with polyhydramnios before and after the conservative management, hence RA PI could not be used as a marker of polyhydramnios evaluation (19).

**Table 1:** Methodological characteristics and patient selection in included studies


gray!50 **Author, year (Ref)**	**Study design**	**Sample size (case/ control)**	**Inclusion criteria**	**Gestational age (weeks)**	**Relevant index**
**Mitra ** ***et al.*** **, 1995 (9)**	Prospective	110	Uncomplicated pregnancies	19-40	RI
**Ozkan ** ***et al.*** **, 2014 (10)**	Prospective	66/60	Isolated oligohydramnios	35-40	RI
**Scott ** ***et al.*** **, 2000 (11)**	Prospective	14	Unexplained oligohydramnios	20-36	PI
**Benzer ** ***et al.*** **, 2015 (12)**	Prospective	300	Low-risk pregnancies	22, 28, 34	RI, PI
**Selam ** ***et al.*** **, 2000 (13)**	Cross-sectional	38	Post-term pregnancies	> 41	PI
**Oz ** ***et al.*** **, 2002 (14)**	Cross-sectional	147	Post-term pregnancies	> 41	RI
**Veille ** ***et al.*** **, 1993 (15)**	Cross-sectional	50	Post-term pregnancies	> 40	S/D
**Sahin ** ***et al.*** **, 2018 (16)**	Cross-sectional	107/323	Borderline amniotic fluid index	34-37	-
**Budunoglu ** ***et al.*** **, 2019 (17)**	Cross-sectional	45/65	Isolated oligohydramnios	25-40	-
**Madazl ** ***et al.*** **, 2021 (18)**	Prospective	25/25	Isolated oligohydramnios	24-37	-
**Figueira ** ***et al.*** **, 2015 (7)**	Prospective	62	Low-risk pregnancies	16-19	-
**Seravalli ** ***et al.*** **, 2019 (3)**	Retrospective	146	Low and high-risk pregnancies	17-38	vcRA-PI
**Akdogan ** ***et al.*** **, 2015 (19)**	Prospective	39	Polyhydramnios	26-36	-
**Yoshimura ** ***et al.*** **, 1997 (20)**	Cross-sectional	39/100	FGR	36-40	PI
**Manabe ** ***et al.*** **, 1995 (21)**	Cross-sectional	7/13	FGR	15-40	PI
**Stigter ** ***et al.*** **, 2001 (22)**	Prospective	16	FGR	26-35	PSV
**Tanabe ** ***et al.*** **, 1992 (23)**	Cross-sectional	32	FGR	28-38	-
**Benavides-Serralde ** ***et al.*** **, 2011 (24)**	Cross-sectional	72	FGR	20-36	-
**Contag ** ***et al.*** **, 2021 (25)**	Retrospective	92	FGR	Last scan	-
**Adıyaman ** ***et al.*** **, 2020 (26)**	Prospective	76/51	Late-onset FGR	32-37	Renal volume
**Silver ** ***et al.*** **, 2003 (27)**	Prospective	34/43	FGR	27-41	Renal volume
**Bates ** ***et al.*** **, 1992 (28)**	Cross-sectional	29	Renal tract dilation	Not determined	-
**Gudmundsson ** ***et al.*** **, 1991 (29)**	Prospective	17	Hydronephrosis	26-39	-
**Wladimiroff ** ***et al.*** **, 1993 (30)**	Retrospective	21, 4	Hydronephrosis, multicystic kidney disease	16-37	PI
**Kaminopetros ** ***et al.*** **, 1991 (31)**	Cross-sectional	11	Unilateral multicystic kidney disease	19-34	PI
**Iura ** ***et al.*** **, 2005 (32)**	Prospective	15 (6, 9)/46	Polycystic kidney, hydronephrosis	20-40	PSV
FGR: Fetal growth restriction, RI: Resistive index, PI: Pulsatility index, S/D: Systolic/diastolic ratio, vcRA-PI: Volume corrected renal artery pulsatility index, PSV: Peak systolic velocity					

## 5. RAD in the assessment of FGR

Chronic hypoxia, because of poor placental perfusion redistributes cardiac output from the periphery to the brain. Vascular resistance in RAs increases while renal blood flow decreases. It is proposed that oligohydramnios in growth-restricted fetuses is caused by reduced urine output owing to decreased renal perfusion in chronic hypoxemia (17). There are conflicting observations about RAD and FGR in literature. First, we have summarized the studies that found some relationship and then the articles with results of no association between RA indices and FGR (Table I).

In a study assessing the association between the blood flow changes and the quantity of amniotic fluid, PI values of the umbilical artery and RA were significantly higher in normal growth fetuses with oligohydramnios in comparison to fetuses with insufficient amniotic fluid. The PI values of the umbilical artery and RA were significantly higher in growth-restricted fetuses with oligohydramnios than in fetuses with sufficient amniotic fluid while the PI of the middle cerebral artery was significantly lower. In addition, a significant negative relationship between the PI value of the RA and the deep vertical pocket, and the AFI was seen (20). An article evaluating changes in vascular resistance of arteries with advancing gestation in normal and growth-restricted fetuses showed the RA-PI values in growth-restricted fetuses were higher than those in normal near-term fetuses (21). However, in another study, the RA-PI did not demonstrate any relationship with cord blood pH, birth weight, or AFI corrected for gestational age. However, PSV showed a marked decrease with time and a significant association with umbilical cord venous pH at delivery and birth weight. They pointed out that the redistribution of the blood flow happens with worsening of the fetal status in the growth-restricted preterm fetus, and this is shown by changes in PSV but not the PI of the fetal RA (22).

In a survey on fetuses who had high RI indices in the umbilical artery and low RI in the fetal middle cerebral artery, the PI values of RA were normal in most of them. Also, the PI values of RA were normal in most of the fetuses with abnormal variability in non-stress test. They suggested that fetal renal blood flow does not vary in chronic hypoxic conditions and assumed that the fetal renal blood flow is adjusted by an autoregulation mechanism (23). In another survey, the peripheral blood in the RA did not show any change in groups with normal or disturbed umbilical artery PI, and AFI showed late alteration (24). In a study on FGR fetuses found in the last scan before delivery, fetal RA resistance did not increase with short-term complications. They did not find the RA-PI value a worthy clinical tool to diagnose a fetus at risk or to understand the delivery time. This finding can be due to adapting to growth restriction conditions (25).

In a study assessing renal volumetric alterations in addition to RAD in growth-restricted fetuses with normal amniotic fluid in comparison to uncomplicated pregnancies, bilateral RAD indices demonstrated no difference in the 2 groups but right, left, and mean renal volume of the fetuses with FGR were lower than the normal group with a significant difference. They explained this difference is not because of decreased blood flow through redistribution but other reasons such as glomerular apoptosis (26).

In an earlier study, measurements of renal volume and RAD blood flow were compared between fetuses with and without FGR. Renal volume in the intrauterine growth-restricted fetuses was less after an adjustment for gestational age. No differences were observed in the RAD measurements. FGR appeared to be linked with a lower fetal renal volume. Because renal volume can be a marker of nephron number, this study supported the thesis that FGR may contribute to congenital oligo nephropathy and may lead to hypertension in future (27).

## 6. RAD in fetal renal diseases

Ultrasound is used to evaluate fetal renal diseases, mainly cysts or morphological changes. Moreover, quantifying the urine bladder and amniotic fluid are often used to evaluate the function of fetal kidney. In addition, ultrasonic color Doppler and pulse Doppler studies can analyze the hemodynamics of the fetus, and indices such as PI and RI were applied to assess the function of fetal kidney (33). There are a few studies about Doppler in renal diseases in literature with inconsistent results. Noticing all of them had a small number of cases, their clinical value is not determined (Table I).

Some of these studies have hypothesized that in obstructive uropathy, the pressure within the pelvicalyceal system and the resistance of the peripheral vasculature of the kidney increases; moreover, evaluating cases of fetal renal tract dilation in one article showed the PI was not significantly changed (28). In another survey, of fetuses with hydronephrosis in the third trimester of pregnancy, no relationship was found between the degree of dilatation and PI (29). However, a study on fetuses with unilateral or bilateral hydronephrosis had shown that the PI was significantly higher in severe hydronephrosis compared with mild hydronephrosis, although the measurements were in the normal range. Regarding multicystic kidney disease, the last-mentioned article showed that the RA-PI of the involved kidney was higher than that on the contralateral normal side (30). In another study, in all fetuses with unilateral multicystic kidney disease, the same result was seen about RA-PI of the involved kidney and the contralateral normal kidney. A significantly higher mean PI in the arteries of the affected kidneys was seen (31).

An article on fetuses with renal disease (polycystic kidney or hydronephrosis) showed that the PSV of the RA in fetuses with renal disease correlates with fetal kidney function, but no significant changes were observed in PI and RI. They supposed that RA peak systolic blood flow waveform of 1.5 standard deviation can be considered as a lower limit of the normal range and seems to be important in the outcome (32).

## 7. RAD in the assessment of monochorionic twin pregnancies

There is a prospective case-control study on women with monochorionic twin pregnancies with and without twin-to-twin transfusion syndrome and the application of RAD. The authors of this article compared RAD measurements between donors and controls, as well as recipients and controls. RA-PI and RI were not different in donors and controls. Donor RA-PSV was significantly lower than what was seen in controls. Comparing recipient twins with controls, no difference was seen in any of the Doppler indices. Among donors in survivors of laser therapy, prelaser RA-PSV ratios were significantly higher than post-laser measurements. This shows that laser therapy makes renal blood flow normal (34).

## 8. Fetal RAD in preeclampsia

Assessment of fetal RA impedance, in appropriately grown fetuses of pregnancies complicated by preeclampsia and a control group of uncomplicated pregnancies, showed that fetuses of mothers with preeclampsia were more likely to have lower RA Doppler S/D ratio and lower RI. They suggested that in such pregnancies with elevation in total peripheral resistance, abnormal RA resistance indices might be associated with renal dysfunction (35).

## 9. Fetal RAD in gestational diabetes mellitus (GDM)

In an article on women with GDM and normal controls, fetal hemodynamic indices of RA and fetal growth indices were collected. RA-S/D, PI, and RI had a positive correlation with birth weight in GDM, but there were no correlations in normal controls (36).

In another study on pregnancies with GDM, weekly Doppler ultrasound was done in the late pregnancy period. Fetal growth indices were also recorded and compared. The GDM group showed significantly higher resistance in RA indices in comparison to the controls. Also, an inverse relationship between umbilical and RA indices and birth weight was seen in the GDM group. In the control group, only umbilical artery PI but not RA showed an inverse relationship with birth weight. The authors supposed that using Doppler indices of the RA in late pregnancy in GDM patients can help in the detection of fetuses with a risk of small for gestational age even when they are in normal range weight (37).

## 10. Prediction of adverse neonatal outcomes

A recent report on 564 women, investigating RAD indices to diagnose fetuses at risk for adverse neonatal outcomes, demonstrated that against their supposition that RA resistance is more sensitive, the RA-PI or RA-PSV were not better than the cerebroplacental ratio or umbilical artery PI in diagnosing fetuses with neonatal complications (involving intensive care unit admission, mechanical ventilation, fetal or neonatal death, intraventricular hemorrhage, respiratory distress syndrome, hypoxic-ischemic encephalopathy, or necrotizing enterocolitis) (38).

## 11. Fetal RAD in evaluating the safety of drugs in pregnancy

Fetal RAD has been used in several clinical trials for evaluating the safety of drugs in pregnancy as a parameter of fetal hemodynamics.

An article comparing intravenous atenolol and pinolol in patients suffering from pregnancy-induced hypertension showed a PI decrease in fetal RA with atenolol and preferred pinolol because of the effects of atenolol on fetal hemodynamics (39). Studies on drugs for the treatment of preterm labor such as nifedipine, sulindac, terbutaline, and indomethacin also used Doppler indices including RA, and saw no significant differences before and after treatment (40-42). Studies analyzing hemodynamic modifications after antenatal corticosteroid treatment did not report any changes in Doppler of investigated vessels including RA (43-45). There are same articles about evaluating the safety of misoprostol, PGE2, and aspirin in literature with the same results (46-48). An article about the effect of maternal cocaine abuse on renal arterial flow reported significantly higher RA-RI and a decrease in the hourly urine output in cocaine-exposed fetuses (49).

## 12. Discussion

This review shows that assessing RA can add value to Doppler assessment in pregnancy. This may be of specific importance in predicting oligohydramnios in the early stages of a pathological condition. The specific role in assessing other issues like FGR could not be demonstrated with the available literature, and the umbilical artery is yet the best parameter to study in high-risk pregnancies such as FGR. Discussing the physiology of fetal kidney function may help to explain different results observed in different studies about the role of RAD in fetal assessment. Fetal RAs are high in resistance and receive a very low fraction of cardiac output in the third trimester (50).

The complexity of the kidney vascularization pattern can be because of variable hemodynamic responses to stimuli and different mechanisms that regulate kidney function (sympathetic nervous system, arginine-vasopressin system, level of angiotensin, levels of prostaglandins, and blood pressure). In assessing renal hemodynamics, on the one hand glomerular filtration is dependent on several factors including hydrostatic filtration, oncotic pressure, capillary permeability, total area constituency, and capillary glomerular tissue, and on the other hand kidney flow is dependent on cardiac blood reserve, vascular tone of renal interstitial, glomerular resistance, and tubular reabsorption. Including all these parameters in a study is almost impossible. In addition, centralization in response to chronic stress is not a simple event and the mechanism involves redistribution of blood among organs (51).

Another note to point out is that RAD assessment is somehow technically difficult due to fetal position, movements, and breathing, especially in third-trimester fetuses, and most of the present studies assessed small populations.

Despite the potential pathophysiology described before it seems there is not enough evidence for introducing RAD as a certain tool in managing high-risk pregnancies in practice. More research is needed to evaluate the effectiveness of RAD in clinical management specifically considering cut-off values and outcomes of interest indicated in the present review. It might give valuable information in diabetic pregnancies with polyhydramnios that oligohydramnios as a sign of hypoxia may manifest later.

## 13. Conclusion

It seems the application of RAD in fetal ultrasound can be useful in assessing some groups like diabetic pregnant women, but it should be used for accompanying other related factors like kidney size to get better results. Further research is needed to evaluate the effectiveness of RAD in clinical management.

##  Conflict of Interest

The authors declare that there is no conflict of interest.
